# Lack of Specific Immune Response after Five Doses of mRNA SARS-CoV-2 Vaccine in a Patient with CD4^+^ T-Cell Lymphopenia but Preserved Responses to CMV

**DOI:** 10.3390/vaccines12040386

**Published:** 2024-04-06

**Authors:** Trinidad Alba-Cano, Eduardo Fernández-Cruz, Roberto Alonso, Sara Muñoz-Gómez, Rebeca Pérez de Diego, Elena García Martínez, Paloma Sánchez-Mateos, Joaquín Navarro Caspistegui, Mónica Martín López, Juana Gil-Herrera

**Affiliations:** 1 Division of Immunology, Hospital General Universitario “Gregorio Marañón”, 28007 Madrid, Spain; trinidad.alba@salud.madrid.org (T.A.-C.); eduardo.fernandezcruz@salud.madrid.org (E.F.-C.); smunozgomez@salud.madrid.org (S.M.-G.); egarciamartinez2@salud.madrid.org (E.G.M.); joaquin.navarro@salud.madrid.org (J.N.C.); monica.martin.lopez@salud.madrid.org (M.M.L.); 2 Instituto de Investigación Sanitaria Gregorio Marañón (IiSGM), 28007 Madrid, Spain; 3 Department of Clinical Microbiology and Infectious Diseases, Hospital General Universitario Gregorio Marañón and CIBER (Centro de Investigación Biomédicas en Red) de Enfermedades Respiratorias, CIBERES, 08028 Barcelona, Spain; roberto.alonso@salud.madrid.org; 4 Laboratory of Immunogenetics of Human Diseases, Innate Immunity Group, IdiPAZ Institute for Health Research, La Paz Hospital, 28046 Madrid, Spain; rpdediego@salud.madrid.org; 5 Department of Immunology, Ophthalmology and ENT, School of Medicine, Universidad Complutense, 28040 Madrid, Spain; paloma.sanchezmateos@salud.madrid.org

**Keywords:** inborn errors of immunity, IEIs, CD4^+^ cytopenia, COVID-19, SARS-CoV-2, CMV, vaccination, IgG response, T-cell response, immunogenicity

## Abstract

Immunogenicity of SARS-CoV-2 mRNA vaccines is highly heterogeneous in patients with inborn errors of immunity (IEIs). This case report analyzes the immune response to mRNA COVID-19 two-dose primary vaccination followed by three boosters in an IEI patient with marked CD4^+^ T-cell cytopenia and diminished thymic output, in comparison with that raised against latent, chronic cytomegalovirus (CMV) infection. Serum IgG antibodies anti-spike (S) protein of SARS-CoV-2 and anti-CMV were both determined by chemiluminescent microparticle immunoassays (CMIAs). SARS-CoV-2 and CMV memory CD4^+^ T-cell responses were simultaneously evaluated in vitro using an activation-induced marker (AIM) assay via multicolor flow cytometry. Throughout the 2-year follow-up that included the administration of five doses of SARS-CoV-2 mRNA vaccines, cellular anti-SARS-CoV-2-specific responses remained consistently negative, with extremely weak humoral responses, while the patient showed in vitro persistent CD4^+^ T-cell reactivity to CMV peptides and high-IgG CMV-specific titers. The assessment of immune responses to vaccines and prevalent viruses is essential in IEI patients in order to take adequate preventive measures.

## 1. Introduction

Vaccines against SARS-CoV-2 have been the most effective measure against the COVID-19 pandemic [1,2]. Some patients with inborn errors of immunity (IEIs) (like Good syndrome, autoimmune polyendocrinopathy candidiasis ectodermal dystrophy (APECED)/anti-IFN autoantibodies and antibody deficiencies with autoimmune/inflammatory disorders) belong to a small group that is highly vulnerable to severe courses of COVID-19 [3,4,5], so IEI patients were prioritized for prompt and repeated SARS-CoV-2 vaccination in our country [6]. Quantitation of vaccine spike (S)-specific serum IgG titers and T-cell assays are very useful as immune-monitoring tools for identifying vaccination efficacy/inefficacy in IEI patients, helping to establish their individualized management [7,8,9,10]. Therefore, 115 IEI patients from our National Reference Unit for Immunodeficiency cohort were enrolled for such humoral and cellular immune monitoring after primary SARS-CoV-2 vaccination. In this study, specific IgG and CD4^+^ T-cell immune responses against cytomegalovirus (CMV) were also tested at the same time, as controls in the laboratory assays [11] and for a better understanding of the immune status of our IEI patients. 

Here, we report the case of one of our SARS-CoV-2 non-responder IEI patients, who showed a strikingly discordant immunogenicity against SARS-CoV-2 neoantigens as compared to CMV immune responses. These findings were confirmed throughout his entire follow-up involving the administration of five doses of mRNA SARS-CoV-2 vaccines.

## 2. Case Report and Methods

A 56-year-old male has attended our Outpatient Clinical Immunology Unit since 2015 because of CD4^+^ T lymphopenia and recurrent infections with inflammatory bowel disease, autoimmunity, very low circulating recent thymic emigrants, increased TCRγ/δ^+^ lymphocytes and oligoclonal TCR rearrangements, as summarized in Table 1. However, he had normal serum levels of all IgG, IgA and IgM isotypes and IgG subclasses, with a positive specific IgG response following 23-valent pneumococcal polysaccharide vaccination (Table 1). He was found to be a carrier of five heterozygous missense variants detected by whole exome sequencing; three were located in two genes phenotypically related to common variable immunodeficiency and two variants were in the *BTNL2* gene associated with susceptibility to Crohn’s disease [12].

**Figure 1:**
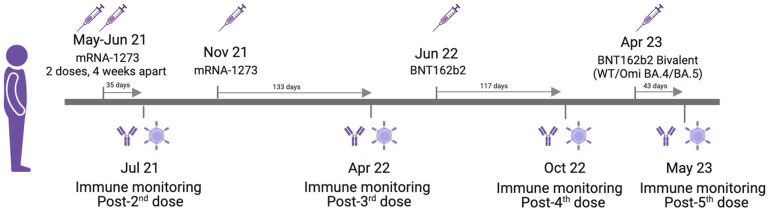
SARS-CoV-2 vaccination schedule and patient’s follow-up. Figure 1 was created by using the free tool BioRender.com.

From 2021 and over a 2-year period, this patient received a primary double dose—28 days apart—of mRNA-1273 vaccine (Moderna, Cambridge, MA, USA), followed by the same Moderna injection in the third dose. As a fourth dose, he received a heterologous booster vaccination with BNT162b2 (Pfizer, New York, NY, USA), and the fifth consisted of the Pfizer BioNTech bivalent vaccine including mRNA from original and Omicron BA.4/BA.5 strains, as depicted in Figure 1. All vaccine doses were well tolerated with no adverse effects. As far as we know—based on clinical symptoms plus SARS-CoV-2 RT-PCR laboratory tests or at-home antigen self-tests with negative results—he has not experienced any active COVID-19 breakthrough.

The healthy control was a 64-year-old male, the relative of a healthcare worker from our division, who received four doses of SARS-CoV-2 vaccines. By the time of his immunological testing, he was naïve to SARS-CoV-2 infection according to antigen tests with negative results. 

The IEI cohort included 115 patients from 22 to 80 years old with a median [P5–P95] age of 53 [26–73] years; 70 are female and 45 are male. The majority predominantly have antibody deficiencies (48 common variable immunodeficiency (CVID) phenotype, 53 isotype or functional deficiencies, 3 agammaglobulinemia and 2 hyperIgM syndromes); 3 patients have combined immunodeficiencies; 3 have complement deficiencies; 2 patients classified as having diseases of immune dysregulation and 1 has a phagocytic disorder. All of them were vaccinated with mRNA SARS-CoV-2 vaccines. 

Detection of serum IgG antibodies against SARS-CoV-2 S protein was performed by a quantitative chemiluminescent microparticle immunoassay (CMIA) using the SARS-CoV-2 IgG II Quant reagent kit on an ARCHITECT i2000 instrument (Abbott; Chicago, IL, USA). The results are expressed as Arbitrary Units per milliliter (AU/mL) and were converted to standardized Binding Antibody Units per milliliter (BAU/mL) using the conversion coefficient provided by the manufacturer (1 BAU = 0.142 × AU). Results above 7.10 BAU/mL were considered positive according to the manufacturer’s indications [13]. 

CMV-specific IgG antibodies were determined using a commercial indirect enzyme immunoassay, the qualitative/semi-quantitative CMIA, on serum samples with the Alinity i analyzer (Abbott, Chicago, IL, USA). The resulting chemiluminescent reaction is measured as relative light units (RLUs) and changed to AU/mL by comparing sample RLUs with those from six quantified standards in a calibration curve. The cut-off considered by the assay is set at 6.0 AU/mL (<6.0 nonreactive, ≥ 6.0 reactive). The measuring interval of the CMV IgG assay is 1.1 to 250.0 AU/mL. Samples with values above the higher limit can be run diluted in order to obtain the real sample values.

An activation-induced marker (AIM) assay by multicolor flow cytometry was used to assess in vitro viral antigen-specific memory CD4^+^ T-cell responses. Briefly, peripheral blood mononuclear cells (PBMCs) were isolated by density gradient centrifugation and re-suspended at a concentration of 10 × 10^6^ cells/mL in TExMACS medium with stable glutamine (Miltenyi Biotec, Bergisch Galdbach, Germany) supplemented with 10% fetal bovine serum (FBS, Sigma-Aldrich, Saint Luois, MO, USA). Then, 100 μL of PBMCs (1 × 10^6^ cells per well) was plated in 96-well U-bottom plates (Corning Inc., New York, NY, USA). An amount of 1 μL of co-stimulatory monoclonal antibodies CD28/CD49d [clones L293/L25] was added to every well. As a positive control condition, 4 μL of anti-human CD3/CD28/CD2 antibodies (ImmunoCult™ Human CD3/CD28/CD2 T Cell Activator, STEMCELL Technologies, Vancouver, BC, Canada) was added. The negative control condition was only supplemented with a volume equivalent of TExMACS medium. An amount of 2 µL of peptide pool compounds of 15-mer peptides with 11–amino acid overlap was used for antigen stimulation conditions (Miltenyi Biotec, Bergisch Galdbach, Germany). In the case of CMV, the pool covers the complete sequence of pp65 protein; for SARS-CoV-2, it covers the immunodominant sequence domains of S protein from original and Omicron strains. These were included in separate wells. Then, cells were incubated for 44–48 h at 37 °C in a humidified atmosphere containing 5% CO_2_. Following incubation, cells from each well were individually collected and washed with 1 mL of PBS/EDTA buffer. Afterwards, lymphocytes in every tube were stained by adding a mix of monoclonal antibodies (mAbs): anti-CD25 APC [clone M-A251] and -OX40 (CD134) PE [clone 134-1] from the CD4 T-cell activation kit (Act-T4 Cell^TM^ Kit), which were used to identify AIMs; another two mAbs anti-CD3 PerCP-Cy5.5 [clone UCHT1] and -CD4 FITC [clone RPA-T4]—also contained in this commercial kit—plus 1 μL of anti-CD45RA APC-H7 [clone HI100] and 1 μL of anti-CD27 PE-Cy7 [clone Clone M-T271] to identify the different memory subsets of CD4^+^ T cells; and 3 µL of PBS. All the reagents were from BD Biosciencies. Cells were incubated for 20 min at room temperature in the dark, washed again and re-suspended in 200 μL of PBS. Finally, cells were acquired in a FACSLyric flow cytometer (BD Biosciences, Franklin Lakes, NJ, USA). At least 100,000 lymphocytes were recorded per sample. Flow cytometry data were analyzed with BD FACSuite software, version 1.5.

**Figure 2:**
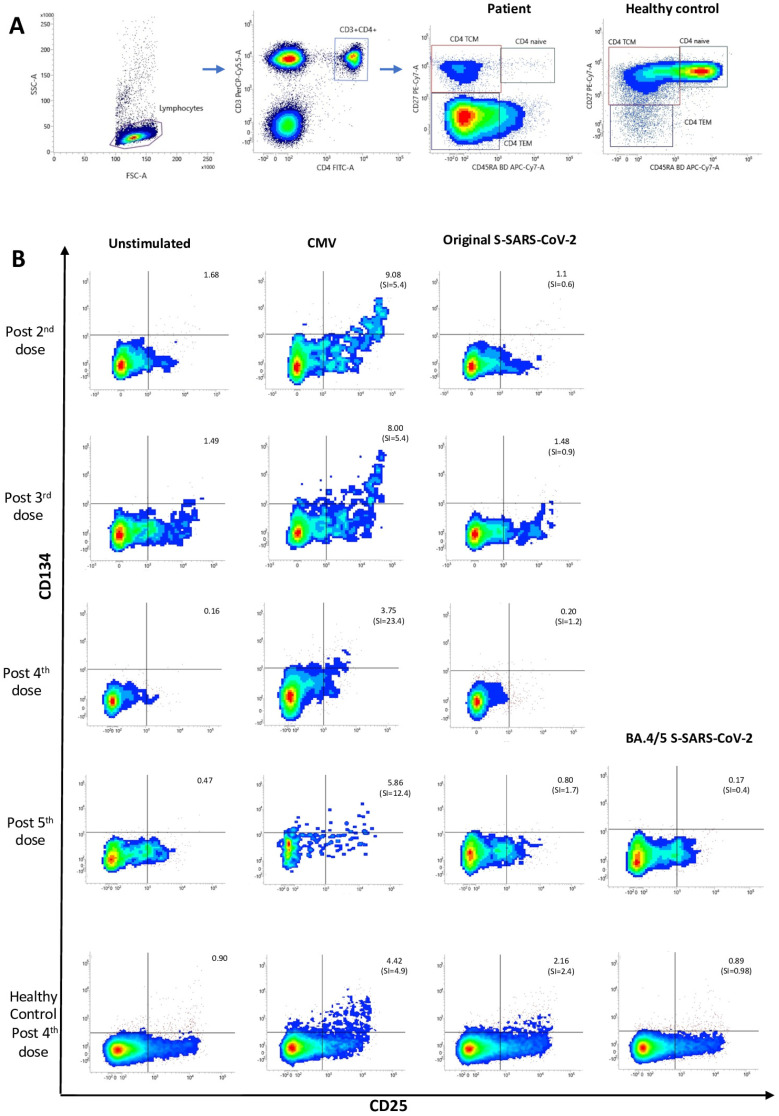
(**A**) Gating strategy and distribution of CD4^+^ T-cell memory subsets in the patient and healthy control. Lymphogate was drawn based on forward and side scatter characteristics, then helper T cells were selected as those lymphocytes coexpressing CD3 and CD4 molecules. Finally, CD45RA and CD27 surface markers were used to determine the different memory cell subsets within CD4^+^ T lymphocytes: TCM (T central memory): CD4^+^CD45RA^−^CD27^+^ T cells, TEM (T effector memory): CD4^+^CD45RA^−^CD27^−^ T cells, naïve: CD4^+^CD45RA^+^CD27^+^ T cells. (**B**) Antigen-specific central memory CD4^+^ T-cell (TCM) responses to CMV and SARS-CoV-2 (original and Omicron BA.4/BA.5 strains). Stimulation index (SI) indicates the fold increase of % CD134^+^CD25^+^ TCM after antigen stimulation over unstimulated condition. Cellular response is considered positive when SI ≥ 2.

The gating strategy of central memory CD4^+^ T cells (TCMs) CD45RA^−^CD27^+^ is shown in Figure 2A. Results are expressed as the stimulation index (SI) calculated by dividing the percentage of AIM+ (coexpressing CD25 and CD134 (OX40)) TCMs after peptide stimulation by the percentage of AIM+ TCM from non-stimulated PBMC (Figure 2B). SI ≥ 2 is considered positive [14]. 

## 3. Results

Table 2 shows no S-specific IgG response after the two initial doses of the mRNA-1273 Moderna vaccine and extremely low titers following the three subsequent boosters, with a very weak trend reaching a maximum titer of 76 BAU/mL after the fifth dose. Following two-dose primary vaccination, the median [P5–P95] of anti-SARS-CoV-2 IgG antibodies was 1040 [186–4803] BAU/mL in our healthy control group (*n* = 38) and 703 [0–5093] BAU/mL in the IEI cohort. However, during the same period of immune monitoring, consistent titers of anti-CMV-specific IgG antibodies were detected, with most of the values above the normal range from our group of 30 healthy CMV-seropositive control individuals (median [P5–P95]: 135.4 [66.4–491.3] AU/mL). Moreover, CMV-specific IgG titers showed a 6-fold increase between the first and second determinations and a gradual slope towards the end of this follow-up.

As shown in Figure 2A, the patient and healthy control exhibited very different memory CD4^+^ T-cell profiles. An increase in the effector memory T-cell (CD45RA^−^CD27^−^) population, with very few naïve T cells (CD45RA^+^CD27^+^), is observed in the patient, while the central memory T-cell subset (CD45RA^−^CD27^+^) is clearly more represented in the healthy control.

Regarding the dynamics of cellular responses, circulating CMV-specific CD4^+^ T cells were detected within the central memory subset (CD45RA^−^CD27^+^) throughout the follow-up (Figure 2B). On the other hand, the patient’s SARS-CoV-2 S-specific CD4^+^ T-cell memory responses were found to be always negative (Figure 2B), including those to original and Omicron variants tested after the fifth bivalent vaccine. In the healthy control, positive memory CD4^+^ T-cell responses were obtained against both CMV and S-SARS-CoV-2 peptides.

## 4. Discussion

The patient reported on here showed a completely negative IgG response to SARS-CoV-2 after the second dose of mRNA-1273 Moderna vaccine. Among our IEI cohort of 115 patients, 12 other patients (CVID (*n* = 6), functional antibody deficiencies (*n* = 2), 1 agammaglobulinemia, 1 Good syndrome, 1 autoimmune lymphoprolipherative syndrome (ALPS) with agammaglobulinemia and 1 APECED) showed absent humoral responses; therefore, 13 out of 115 IEI patients were found to be non-responders.

The patient showed even lower production of IgG-specific anti-SARS-CoV-2 when compared with four different Danish cohorts of immunosuppressed patients (solid organ transplantation recipients) who were also studied after the second, third, fourth and fifth doses of mRNA vaccines against COVID-19 [15]. Regarding the general population, quantitative SARS-CoV-2 IgG levels were indeed lower than those from the health worker group of similar age at our hospital, tested after the two initial doses of mRNA COVID-19 vaccination by using the same CMIA methodology [13], and also below the 2.5th percentile of 627 Chinese individuals after receiving two doses of inactivated SARS-CoV-2 vaccine from Sinopharm [16]. Such antibody generation failure correlates with repeatedly undetectable CD4^+^ T-cell responses in vitro at any point in our patient´s follow-up. In contrast, this SARS-CoV-2 non-responder patient was able to produce high titers of specific serum IgG anti-CMV in vivo and showed positive CMV-specific T-cell responses in vitro. The lack of humoral response to SARS-CoV-2 also contrasts with the previous demonstration of a good specific IgG antibody production following other immunization schedules [12].

Absent cellular responses to SARS-CoV-2 even after the fifth vaccine dose in this patient could be explained in the context of his T-cell immunodeficiency with CD4^+^ cytopenia (CD4^+^ T-cell counts < 250/µL throughout his long-term follow-up in our Clinical Immunology Unit, lasting 8 years; not shown), and it is in agreement with the decreased CD4^+^ T-cell counts (<500/mm^3^) found in healthy Chinese individuals with no seroconversion after the two initial doses of SARS-CoV-2 vaccine [16].

A low recent thymic output with oligoclonal TCR rearrangements and a limited T-cell repertoire [17] is associated with impaired response to antigens and high risk of developing autoimmune diseases or cancer [18]. In this case, the failure of SARS-CoV-2 immunodominant S-peptide epitopes to elicit a thymus-dependent response could be due—at least in part—to the oligoclonal nature of his TCR repertoire [17,18]. On the other hand, the lack of response to SARS-CoV-2 neoantigens in this patient does not fit with the response found by other authors who have described the generation of SARS-CoV-2-specific CD4^+^ T-cell responses from cross-reactive CMV-specific T cells after stimulation of pre-pandemic cryopreserved PBMC with SARS-CoV-2 peptides in CMV-seropositive donors [19].

The particular biased CD4^+^ T-cell memory phenotype shown by our patient can be seen in other IEI patients [20,21,22] and could as well be related to persistent CMV infection with expansion of CMV-specific effector and memory cells [20,23,24,25]. Such *inflation* of CMV-specific T cells [26] along with a substantial loss in the relative proportion of naïve T lymphocytes in CMV-infected individuals [25,27,28] may additionally contribute to the inefficient SARS-CoV-2 immunization in the patient presented here.

The knowledge of this particular patient´s immune status regarding CMV is indeed relevant because of the theoretical risk of subsequent viral reactivation from latency associated with persistent low CD4^+^ T-cell counts [29,30]. CMV enteritis and colitis are frequently observed in immunocompromised patients with severe depletion of CD4^+^ T cells [31]. In our patient, the anti-CMV IgG curve would suggest a CMV reactivation [32], although it would be subclinical since no significant clinical changes were observed by the time of the curve peak. Chanouzas et al. [33] have reported a significant increase in anti-CMV IgG antibodies associated with subclinical reactivation episodes and impaired immune responses to the 13-valent pneumococcal conjugate vaccine in patients with vasculitis. Moreover, the administration of valacyclovir in some of these patients improved their vaccine response [33]. Future studies are needed to determine whether the use of antiviral therapy in CMV-seropositive individuals is beneficial to favoring the immune response in those with poor response or non-responders to SARS-CoV-2 or other vaccines.

With regard to SARS-CoV-2 infections, so far this patient remains apparently naïve. Our concerns about a more severe form of COVID-19 in the future arise not only from his lack of SARS-CoV-2-specific immune responses presented here but also because he displays a number of well-known poor prognostic factors like total lymphopenia, CD4^+^ cytopenia and CMV seropositivity [34,35].

The strikingly different immune responses to SARS-CoV-2 mRNA vaccines when compared to other pathogen immunizations or persistent infections shown in this case report illustrate and confirm that IEI patients need a tailored approach to guide SARS-CoV-2 boosting recommendations and that additional preventive strategies (different vaccine platforms, antiviral prophylaxis…) should be considered in SARS-CoV-2 non-responder IEI patients. 

## 5. Conclusions

The present case highlights the indication of immune monitoring following SARS-CoV-2 vaccination and the benefits of an extensive study of responses to different vaccines or natural infection with other microorganisms in IEI patients. A diversity of underlying factors (decrease in naïve T cells, limited TCR repertoire, CMV latent infection…) alone or in combination may lead to a lack of immunogenicity to the COVID-19 vaccine and should be taken into consideration in non-responder IEI patients. Adequate immune monitoring which includes the evaluation of T-cell immunity (i.e., by AIM expression (CD134, CD25, CD137, CD154), cytokine production (IFNg, IL-2) at the single cell level by flow cytometry or ELISpot or at the population level by ELISA/Quantiferon assays) in addition to the quantitation of specific serum IgG following vaccination campaigns in the IEI context seems essential in order to identify higher-risk patients and use additional strategies to protect these non-responders from severe SARS-CoV-2 infection.

## Figures and Tables

**Table 1:** Immunological characterization of the patient and genetic variants detected by whole exome sequencing.

Laboratory Parameter			Result	Reference Range
**Immunoglobulins**				
IgG (mg/dL)			1460	670–1650
IgA (mg/dL)			104	98–543
IgM (mg/dL)			139	49–302
**IgG subclasses**				
IgG1 (g/L)			7.48	2.78–8.22
IgG2 (g/L)			6.28	1.47–6.29
IgG3 (g/L)			0.62	0.15–1.3
IgG4 (g/L)			0.41	0.032–1.315
**Antigen-specific responses**				
Anti-pneumococcal polysaccharide IgG antibodies (mg/dL)			Pre-vaccination 1.1 Post-vaccination > 27	1–19.1
Anti-cytomegalovirus (CMV) IgG antibodies (AU/mL)			Positive	≥6.0
**Autoimmunity**				
Anti-nuclear autoantibodies			Positive 1/640	<1/80
Anti-dsDNA autoantibodies (IU/mL)			Positive 39	0–15
Anti-histone autoantibodies (relative intensity (%))			Positive 30.9	≥15.9
Anti-cardiolipin IgG autoantibodies (GPL U/mL)			Positive 13.7	0–10
**Lymphocyte subpopulations**				
% T cells CD3^+^ (cells/μL)			69 (396)	55–82 (700–2100)
% helper T cells CD4^+^ (cells/μL)			25 (148)	28–57 (300–1400)
% cytotoxic T cells CD8^+^ (cells/μL)			37 (218)	10–39 (200–1200)
CD4/CD8			0.68	1.00–3.60
% TCR γ/δ+ T cells			18	0.8–11.00
% CD4^+^CD45RA^+^CD31^+^ cells (recent thymic emigrants)			3	14.4–38.3
% CD3^+^CD45RA^+^CD45RO^−^ (naïve T cells)			12	17.6–39.6
% B cells CD19^+^ (cells/μL)			13 (75)	6–19 (100–500)
% CD19^+^IgD^+^CD27^−^ cells (naïve B cells)			59	28–79
% CD19^+^IgD^+^CD27^+^ cells (memory IgM B cells)			28	8–37
% CD19^+^IgD^−^CD27^+^ cells (switched memory B cells)			10	5–35
% NK cells CD56^+^ (cells/μL)			18 (101)	7–31 (90–600)
**Immunogenetics**				
TCR rearrangements			Oligoclonal	
**Gene**	**Dbsnp**	**Genomic Location**	**Protein Change**	**Associated Phenotype**
*IRF2BP2*	rs763707638	g.1:234608652: C > G	p.Glu281Asp	Common variable immunodeficiency (CVID)
*TNFRF13C*	rs77874543	g.22:41926712: G > C	p.Pro21Arg	
	rs61756766	g.22:41925447: G > A	p.His159Tyr	
*BTNL2*	rs115653647	g.6:32403216 C > T	p.Gly143Asp	Crohn’s disease susceptibility
	rs28362680	g.6:32403039 G > A	p.Ala202Val	

Abnormal values are underlined.

**Table 2:** IgG antibodies against SARS-CoV-2 and CMV in immune monitoring.

Vaccine	Anti-SARS-CoV-2 IgG Antibodies (BAU/mL)	Anti-CMV IgG Antibodies (AU/mL)
1^st^ and 2^nd^ doses mRNA-1273 (Moderna)	0	163
3^rd^ dose mRNA-1273 (Moderna)	9.4	1026
4^th^ dose BNT162b2 (Pfizer/BioNTech)	17.8	606
5^th^ dose BNT162b2 bivalent (Pfizer/BioNTech)	76.7	562

## Data Availability

Data are contained within the article.
